# Mutation of G‐protein γ subunit *DEP1* increases planting density and resistance to sheath blight disease in rice

**DOI:** 10.1111/pbi.13500

**Published:** 2020-12-01

**Authors:** Jing Miao Liu, Qiong Mei, Cai Yun Xue, Zi Yuan Wang, Dao Pin Li, Yong Xin Zhang, Yuan Hu Xuan

**Affiliations:** ^1^ Department of Agricultural and Biological Technology Wenzhou Agricultural Science Research Institute (Wenzhou Vocational College of Science & Technology) Wenzhou China; ^2^ College of Plant Protection Shenyang Agricultural University Shenyang China

**Keywords:** DEP1, tiller angle, sheath blight disease, LPA1, rice

One of the important goals of crop breeding is yield improvement. Among the yield indices, the tiller angle is tightly associated with enhancing photosynthetic efficiency and facilitating enhanced planting density (Sakamoto *et al*., 2006; Wang and Li, 2008). Rice plants with erect tillers, leaves and panicles allow a high‐density planting system for high yields but are more susceptible to the occurrence of sheath blight disease causing yield reduction. Therefore, the antagonistic relationship between crop yield and immunity pathways makes crop breeding extremely difficult (Ning *et al*., 2017). In our previous studies, we found that overexpression of loose plant architecture 1 (LPA1) reduced the tiller and lamina joint angle but increased resistance to sheath blight disease through activation of PIN1a‐mediated auxin distribution, suggesting the breeding potential of *LPA1* in high‐density planting systems (Liu *et al*., 2016; Sun *et al*., 2019). To further analyse the mechanism of tiller angle and sheath blight regulation, we performed a yeast two‐hybrid selection and identified G‐protein γ subunit DEP1 (dense and erect panicle 1, Os09g26999) as a novel interactor of LPA1. The heterotrimeric G proteins, comprising α, β and γ subunits, are key players in the transmission of extracellular signals via membrane‐spanning G‐protein‐coupled receptors to intracellular effectors (Gilman, 1987), and panicle erectness is controlled by a dominant allele of *DEP1*, which reduces the length of the inflorescence internode (Huang *et al*., 2009). Further analysis indicated that DEP1 interacted with both full‐length LPA1 and its N‐terminal region (indeterminate domain, IDD) (Figure 1a). Furthermore, coimmunoprecipitation (co‐IP) and split‐GFP assays confirmed that LPA1 interacted with DEP1 in the nucleus (Figure 1b,c).

**Figure 1:**
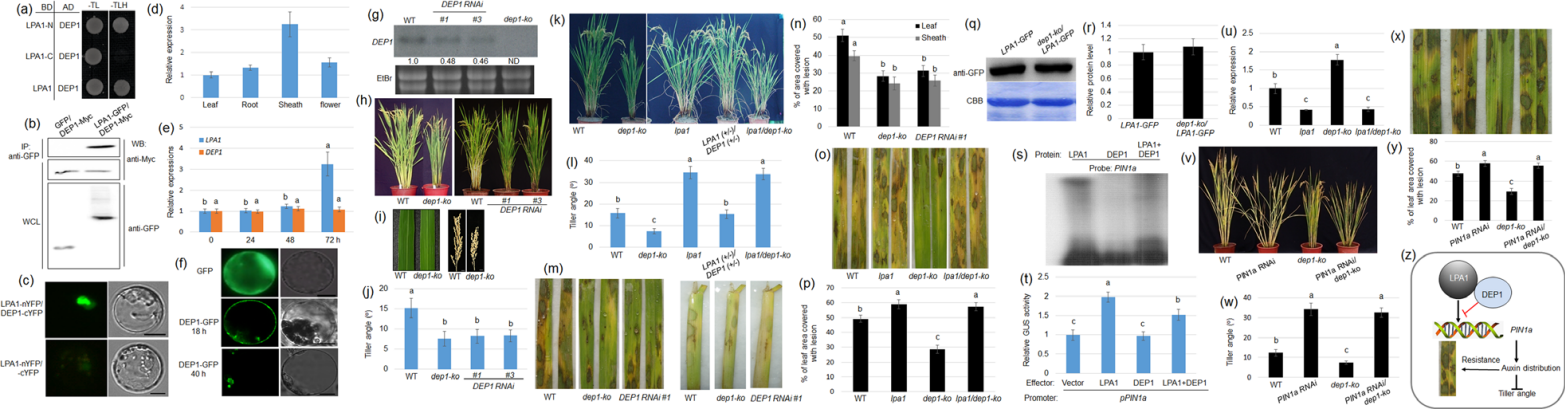
DEP1 controls the tiller angle and resistance to sheath blight disease in rice. (a) The interaction between LAP1 and DEP1 was analysed in a yeast two‐hybrid system. Interaction between activating domain (AD)‐DEP1 and binding domain (BD)‐LPA1 (full length), BD‐LPA1‐N (N‐terminal), or BD‐LAP1‐C (C‐terminal) was analysed. (b) Coimmunoprecipitation (co‐IP) was performed to analyse the interaction between DEP1 and LPA1 in tobacco leaves. DEP1‐Myc + LPA1‐GFP or DEP1‐Myc + GFP were transfected into tobacco leaves. Proteins immunoprecipitated using anti‐GFP were detected using anti‐Myc antibody. The levels of DEP1‐Myc, GFP and LPA1‐GFP from whole‐cell lysates (WCL) were detected using anti‐Myc and anti‐GFP antibodies, respectively. (c) Reconstitution of GFP fluorescence from LPA1‐nYFP + DEP1‐cCFP and LPA1‐nYFP + cCFP. Bars = 10 μm. (d) Expression patterns of *DEP1* in the leaf, root, sheath and flower tissues were examined. Data indicate average ± standard error (SE) (*n* =3). (e) Expressions of *LPA1* and *DEP1* were tested after 0, 24, 48 and 72 h of *R. solani* AG1‐IA inoculation. Data indicate average ± standard error (SE) (*n* =3). (f) Free GFP and DEP1‐GFP signals were detected in the protoplasts. GFP signal was checked at 18 and 48 h. Bars = 10 μm. (g) *DEP1* expression level in wild type (WT), *DEP1 RNAi* lines (*#1* and *#3*) and *dep1‐ko* was analysed by northern blot analysis. EtBr staining was used as a loading control. The numbers shown at the bottom of the blot indicate the relative density of each band. ND: not detected. (h) Shown are 3‐month‐old WT, *dep1‐ko* and *DEP1 RNAi* lines (*#1* and *#3*) plants. (i) The leaf and panicle of WT and *dep1‐ko* were photographed. (j) Tiller angles of plants from panel (h) are shown. Data indicate average ± SE (n> 10). (k) Shown are 3‐month‐old WT, *dep1‐ko*, *lpa1*, *DEP1 (+/‐)/LPA1 (+/‐)* and *lpa1/dep1‐ko*. (l) Tiller angles of the lines from panel (k) are calculated. Data indicate average ± SE (*n* > 10). (m) Leaves and sheath from the WT, *dep1‐ko* and *DEP1 RNAi #1* had been inoculated with *R. solani* AG1‐IA. Each experiment was performed in triplicate. (n) The percentage of lesions in the leaves and sheath shown in (m) was examined. Data indicate average ± SE (*n* > 10). (o) Leaves from the WT, *lpa1*, *dep1‐ko* and *lpa1*/*dep1‐ko* plants were inoculated with *R. solani* AG1‐IA. (p) The percentage of lesions in the leaves from panel (o) was examined. Data indicate average ± SE (*n* > 10). (q) LPA1‐GFP protein levels in *LPA1‐GFP* and *dep1‐ko/LPA1‐GFP* transgenic plants were examined. The proteins stained with Coomassie brilliant blue (CBB) were used as the loading control. (r) The band density shown in panel (q) was calculated. Data indicate average ± SE (*n* =3). (s) An electrophoretic mobility shift assay (EMSA) was conducted to evaluate the affinities of LPA1 and DEP1 to the *PIN1a* promoter. (t) Transient expression of *p35S*:*LPA1* alone or *p35S:LPA1* and *p35S:DEP1* together with a construct including the *GUS* gene under the control of 1.5 kb *PIN1a* promoter in protoplast cells. A luciferase gene driven by the 35S promoter was used as an internal control to normalize GUS expression. Error bars represent ± SE (*n* = 6). (u) Expression level of *PIN1a* in WT, *lpa1*, *dep1‐ko* and *lpa1*/*dep1‐ko* plants. Data indicate average ± SE (*n* =3). (w) Shown are 3‐month‐old WT, *PIN1a RNAi*, *dep1‐ko* and *PIN1a RNAi*/*dep1‐ko* plants. (v) Tiller angles of the lines from panel (u). Data indicate average ± SE (*n* > 10). (x) Leaves from the WT, *PIN1a RNAi*, *dep1‐ko* and *PIN1a RNAi*/*dep1‐ko* plants were inoculated with *R. solani* AG1‐IA and were photographed after infection. (y) The percentage of lesions in the leaves shown in panel (x) was examined. Data indicate average ± SE (*n* > 10). (z) Schematic diagram showing LAP1‐DEP1 regulating *PIN1a* transcription, and its mediated auxin distribution on sheath blight resistance and tiller angle. Different letters above the columns indicate a statistically significant difference between groups. The lesion areas in the leaves and sheath were calculated after 3 days following inoculation.

Through qPCR, we found that *DEP1* expression in sheaths is high compared with that in leaves, roots and flowers (Figure 1d). *Rhizoctonia solani* inoculation induced *LPA1*, but not *DEP1* (Figure 1e), and DEP1‐GFP was localized at the plasma membrane and nucleus (Figure 1f). To analyse *DEP1* function in the *japonica* rice cultivar Dongjin, a *DEP1* knockout mutant *dep1‐ko* (An *et al*., 2003) (PFG_3A‐02648) with the T‐DNA inserted into the first intron, and *DEP1* RNAi lines were used. Northern blot results confirmed that *DEP1* expression was suppressed by about 50% in two *RNAi* lines (*#1* and *#3*) while it was not detected in *dep1‐ko* (Figure 1g). Compared with wild type, *dep1‐ko* and *DEP1 RNAi* plants exhibited a narrow tiller angle, similar shape of leaves and a short panicle (Figure 1h,i,j).

It has previously been shown that *lpa1* causes a wider tiller angle (Liu *et al*., 2016; Wu *et al*., 2013). Further genetic studies showed that *lpa1* plants were similar to *lpa1/dep1‐ko* plants exhibiting a wider tiller angle. However, the tiller angle of plants that were heterozygous for both genes *(LPA1* (+/‐)/*DEP1* (+/‐)) was similar to that of wild‐type plants (Figure 1k,l). In addition, overexpression of *LPA1* has been shown to increase resistance to rice sheath blight (Sun *et al*., 2019). Interestingly, *dep1‐ko* and *DEP1 RNAi* plants were less susceptible to sheath blight compared with wild‐type plants (Figure 1m,n). Upon further examination, we discovered that *lpa1* and *lpa1/dep1‐ko* plants exhibited similar symptoms and were more susceptible, while *dep1‐ko* plants were significantly less susceptible to sheath blight than wild‐type plants (Figure 1o,p).

Even though DEP1 interacts with LPA1, but Western blot analysis showed that LPA1‐GFP protein levels were similar in *LPA1‐GFP* and *dep1‐ko/LPA1‐GFP*, a genetic combination by crossing *LPA1‐GFP* and *dep1‐ko* plants (Figure 1q,r). LPA1 activates *PIN1a* via promoter binding, which increases planting density and resistance to sheath blight disease (Sun *et al*., 2019). Therefore, we further tested the role of DEP1 in LPA1‐mediated *PIN1a* activation via the EMSA and transient assays. EMSA result indicated that DEP1 inhibits the binding of LPA1 to the *PIN1a* promoter (Figure 1s). The transient assay by co‐transformed with *p35S:LPA1*, *p35S:DEP1* or *p35S:LPA1* together with *p35S:DEP1* and a vector expressing the beta‐glucuronidase gene (GUS) under the control of *pPIN1a* promoter in protoplast cells revealed that co‐expression of DEP1 reduced the ability of LPA1 to stimulate the relative GUS activity (Figure 1t), and qPCR results also showed that *PIN1a* expression level was higher in *dep1‐ko* than in *lpa1*, *lpa1/dep1‐ko* and wild‐type plants (Figure 1u). Further genetic studies demonstrated that *PIN1 RNAi* plants were similar to *PIN1 RNAi*/*dep1‐ko* plants exhibiting a wider tiller angle (Figure 1v,w). In addition, *PIN1a RNAi* and *PIN1 RNAi*/*dep1‐ko* plants were more susceptible, while *dep1‐ko* was less susceptible to sheath blight compared with wild‐type plants (Figure 1x,y).

Taken together, our analyses revealed that DEP1 interacts with LPA1 to regulate *PIN1a* expression and that down‐regulation of *DEP1* enhanced planting density by decreasing the tiller angle and at the same time promoted rice resistance to sheath blight disease (Figure 1z). In addition, DEP1 inhibited LPA1‐dependent activation of *PIN1a* transcription via interacts with the N‐terminal region of LPA1, which is the IDD domain region, a known DNA‐binding domain (Kozaki *et al*., 2004). Our data suggest that the interaction between DEP1 and the IDD domain inhibits the DNA‐binding ability of LPA1, thereby suppressing *PIN1a* expression, leading to an increase in planting density and resistance to sheath blight disease in rice.

## Conflict of interests

The authors declare no conflict of interest.

## Authors’ contributions

JML and YHX designed the experiments. JML, QM, CYX and ZYW performed the experiments. DPL and YXZ manipulated plant materials. JML, QM and YHX analysed data. JML and YHX wrote the manuscript. All authors read and approved the final manuscript.
